# Environmental Health Risk Assessment of Dioxin Exposure through Foods in a Dioxin Hot Spot—Bien Hoa City, Vietnam

**DOI:** 10.3390/ijerph7052395

**Published:** 2010-05-14

**Authors:** Tran Thi Tuyet-Hanh, Le Vu-Anh, Nguyen Ngoc-Bich, Thomas Tenkate

**Affiliations:** 1 Hanoi School of Public Health, Hanoi, Vietnam; E-Mail: lva@hsph.edu.vn; 2 Vietnam Public Health Association, Hanoi, Vietnam; E-Mail: nnb@hsph.edu.vn; 3 School of Public Health, Queensland University of Technology, Brisbane, Australia; E-Mail: t.tenkate@qut.edu.au

**Keywords:** environmental health risk assessment, dioxin, Bien Hoa, Vietnam

## Abstract

This study used the Australian Environmental Health Risk Assessment Framework to assess the human health risk of dioxin exposure through foods for local residents in two wards of Bien Hoa City, Vietnam. These wards are known hot-spots for dioxin and a range of stakeholders from central government to local levels were involved in this process. Publications on dioxin characteristics and toxicity were reviewed and dioxin concentrations in local soil, mud, foods, milk and blood samples were used as data for this risk assessment. A food frequency survey of 400 randomly selected households in these wards was conducted to provide data for exposure assessment. Results showed that local residents who had consumed locally cultivated foods, especially fresh water fish and bottom-feeding fish, free-ranging chicken, duck, and beef were at a very high risk, with their daily dioxin intake far exceeding the tolerable daily intake recommended by the WHO. Based on the results of this assessment, a multifaceted risk management program was developed and has been recognized as the first public health program ever to have been implemented in Vietnam to reduce the risks of dioxin exposure at dioxin hot-spots.

## 1. Issue Identification

Bien Hoa City is in the west of Dong Nai Province, approximately 32 km north of Ho Chi Minh City (formerly Saigon), with a total area of 154.67 km^2^ and a population of about 541,495. It is the social and economic center of Dong Nai Province, a focal point in the national transportation system, and one of the most important national industrial areas. Trung Dung and Tan Phong wards are located next to the Bien Hoa Airbase that has been reported as the most severe dioxin hot spot in Vietnam. The population of Trung Dung is 22,524 and they reside in six resident blocks with a total area of 80.75 hectares, while Tan Phong has 34,766 inhabitants within an area of 1,686.16 hectares [1]. Located within the Tan Phong Ward, Bien Hoa Airbase has received substantial attention from national and international environmental groups due to high dioxin contamination levels caused by the spraying of the Agent Orange herbicide during the Vietnam War, and particularly during Operation Ranch Hand. The most toxic compound of dioxin family is 2,3,7,8-tetrachlorodibenzo-p-dioxin or TCDD, which can cause cancer in humans and is classified as a Group I Carcinogen [2]. In addition to cancer, exposure to dioxin is linked to severe reproductive, developmental problems, and many other adverse health impacts [3,4].

During the Vietnam War, approximately 159,000 barrels of herbicides (in which Agent Orange accounted for 98,000 barrels) were transported by the US Army to Bien Hoa Airbase for the Ranch Hand Mission [5]. In order to load herbicides conveniently onto aircraft for aerial spraying, the contents were pumped into large 28,000 liter tanks. It was documented that at least four spills of Agent Orange and Agent White from these tanks occurred between December 1969 and March 1970 [6]. As a consequence, a large amount of herbicide containing dioxin was spilt onto land, causing considerable soil, water, mud and food contamination by dioxin at the Airbase and its vicinities. In addition to the dioxin which was released during the war, dioxin may have also been released into the Bien Hoa environment through the burning of waste at low temperature, the use of pesticides and herbicides in agriculture, and through other industrial uses [6]. However, a previous study has shown that the Agent Orange released during the war has remained the primary source of dioxin in Bien Hoa [6].

Samples of soil, sediment, various types of local foods and blood samples of local residents at Bien Hoa City have been shown to have elevated levels of dioxin [5,7,8]. The estimated amount of contaminated soil at the Bien Hoa Airbase in need of remediation is approximately 70,000 tons, and the cost of this remediation would exceed US $20 million if high temperature approaches were used [9]. Local people, especially those living at Trung Dung and Tan Phong wards are believed to face a range of health risks due to exposure to dioxin in the environment, particularly through consumption of locally-sourced food products. According to Nguyen (Dong Nai Association of Victims of Agent Orange/Dioxin, 2007) an estimated 13,150 people within the Province experience adverse health effects due to ongoing exposure to Agent Orange [1].

Realizing that local residents in the vicinity of Bien Hoa Airbase were facing a range of health risks associated with dioxin exposure, in 2007 the Vietnam Public Health Association (VPHA) together with its branch in Dong Nai Province proposed the implementation of a risk management program to reduce the risk of dioxin exposure for people living in this area. Before developing this intervention, an environmental health risk assessment of dioxin exposure through foods for local residents in Trung Dung and Tan Phong wards, Bien Hoa City was undertaken to provide an appropriate evidence base for developing an effective risk management program.

## 2. Hazard Identification

### 2.1. Dioxin Toxicity

The term dioxin is used to describe a group of 75 chemicals called polychlorinated dibenzodioxins (PCDDs). These mostly come from human sources and persist in the environment for a long period of time [3]. Only 7 of the 75 dioxins have dioxin-like toxicity and they exhibit similar toxic effects caused by binding to a complex molecule known as the aryl hydrocarbon or “Ah” receptor. It is believed that the tighter the binding to the Ah receptor, the more toxic the chemical. The most toxic member of dioxin group is 2,3,7,8-tetrachlorodibenzo-p-dioxin (TCDD), which has the greatest affinity for the Ah receptor, and is considered to be the most toxic chemical produced by humans. Another important factor influencing toxicity level of dioxin compounds is the number of chlorines in the molecule and the positions of attachment of the chlorines. Compounds with four or more chlorines and compounds with chlorines attached at the 2, 3, 7, and 8 positions are particularly toxic. It is thought that the chlorine number and position probably affects the toxicity of the molecules by changing their shape, which in turn determines their ability to bind to the Ah receptor [3].

### 2.2. Physio-Chemical Properties

Dioxin compounds dissolve poorly in water, but well in oils, fats, organic solvents, and therefore adhere strongly to organic components of soil and water. They have a low vapor pressure, and do not evaporate readily. Since they do not react with oxygen or water and are not broken down by microorganisms, they persist in the environment for very long periods of time. Therefore, despite the use of Agent Orange nearly 40 years ago, the levels of dioxin (mainly TCDD) in many hot spots in Vietnam including Bien Hoa Airbase remain very high today [10–13]. Under certain conditions, dioxin is able to be broken down very slowly by sunlight, with the most stable members of the group having four or more chlorines [2,3,14].

### 2.3. Environmental Fate

Once released into the atmosphere, dioxin often binds to other particulates such as incinerator ash. In this case it is shielded from photo-degradation and is able to stay suspended for a long period of time before settling [2]. In regard to dioxin persistence in soil, within the top 0.1 centimeters of surface soil, it has a half life of 9 to 15 years and in subsurface soil (below 0.1 cm) the half live is 25 to 100 years [15]. In water, dioxin accumulates in the bottom mud and sediments of rivers, lakes, and the ocean. In addition, since dioxin is hydrophobic and lipophilic, in water it is taken up readily by aquatic organisms and is concentrated as it moves up the food chain to fish and eventually to humans. For example, dioxin concentration in fish is 100,000 times higher than that in the surrounding environment [3]. A recent study by Schecter *et al.* showed that dioxin levels in some fish samples obtained from the Bien Hoa Market and Bien Hung Lake near Bien Hoa Airbase were very high (e.g., TCDD in fish were from 0.063–65 ppt wet weight) [8]. Dioxin in soil particles or dust attaches to grass, vegetables and crops. Animals that feed on contaminated grass such as cows, buffalo, and goats, and other free ranging animals such as ducks, chicken and wild goose that are raised in areas containing contaminated soil can concentrate dioxin in their meat [3,8]. For example, in the study by Schecter *et al.* (2003), a marked elevation of TCDD was reported in some of the food products, including ducks with 276 ppt and 331 ppt wet weight, chickens from 0.031–15 ppt wet weight, and a toad with 56 ppt wet weight, while the usual TCDD levels in food are less than 0.1 ppt [8]. Normally, plant roots do not take-up dioxin through soil or water, except for some species such as pumpkin and carrot [16,17].

### 2.4. Absorption, Distribution and Excretion

The rate of absorption of dioxin depends on the route of administration, its molecular size and solubility [2]. Tests on mice show that the absorption rate of TCDD through the small intestine and the lungs is between 50% and 90% [19,20]. Dermal absorption is much more limited, probably less than 1% [2,19,20]. Observation of a 42-year old male volunteer who ingested 105 ng of TCDD showed that more than 87% of this dose was absorbed from the gastrointestinal tract [22]. Once TCDD and other compounds in the dioxin family are absorbed in the body, they are readily distributed through the bloodstream to all organs [18]. Because dioxin dissolves poorly in water, it does not dissolve well in the blood, and so stays there for only a short time and tends to accumulate in fatty tissues and in the liver [22]. Schecter *et al.* 2003 reported much higher dioxin levels in lipids than in meat, such as total TEQ for ducks ranged from 286–343 ppt wet weight and 536–550 ppt lipid; for chickens from 0.35–48 ppt wet weight and 0.95–74 ppt lipid, for fish from 0.19–66 ppt wet weight and 3.2–15,349 ppt lipid, and for toad it was 80 ppt wet weight and 11,765 ppt lipid [8]. The body excretes dioxin by first metabolizing or converting it to more water soluble and less harmful compounds in the liver. However, scientific evidence shows that in people and laboratory animals, these processes occur very slowly, and the rate of excretion differs among individuals and species. Half-lives identified have ranged from 11 days in hamsters [18], 17 to 31 days in rats, but less in mice [2,23], and about 391 days in rhesus monkeys [24]. In humans, the half-life reported has been 2,120 days [18] and between 5.8 to 14.1 years [25].

### 2.5. Human Health Impacts

There have been a large number of studies undertaken worldwide to examine the health impacts of dioxin on human, animals and the ecosystem. Studies on animals have show that dioxin exposures result in damage to a number of organs, including the liver, reproductive system, nervous system, immune system, hormonal system, cardio-vascular system, and the lungs [3]. All available evidence indicates that dioxin exposure is associated with cancer in humans in a linear fashion. The International Agency for Research on Cancer (IARC) has classified 2,3,7,8-TCDD as Group I carcinogen, indicating there is no safe dose for dioxin exposure [2].

Further, the Institute of Medicine (2006) after reviewing recent scientific publications on associations between health outcomes and exposure to TCDD and other chemicals in herbicides used in Vietnam noted that “sufficient evidence” exists to link chronic lymphocytic leukemia, soft-tissue sarcoma, non-Hodgkin’s lymphoma, Hodgkin’s disease, and chloracne with exposure. They also concluded that there is “limited or suggestive” evidence of an association between TCDD exposure and laryngeal cancer, cancer of the lung, bronchus, or trachea, prostate cancer, multiple myeloma, AL amyloidosis, early-onset transient peripheral neuropathy, porphyria cutanea tarda, hypertension, Type 2 diabetes (mellitus), and spinal bifida in offspring of exposed people [4].

## 3. Dose-Response Assessment

Studies on effects of dioxin exposure in experimental animals indicate dioxin causes a number of toxic effects, including adverse effects on the liver and skin, on development, and on the reproductive, immune and nervous systems. Table 1 lists some sensitive adverse effects of dioxin and the body burdens estimated to cause those effects in rhesus, rat, and mouse.

Vietnam has not developed a standard or guideline for dioxin levels in the environment. According to standards from some developed countries (e.g., Germany, Japan, America, Netherlands) the acceptable dioxin concentrations in soil of residential areas is 1000 pg/g TEQ (Toxic Equivalents); and between 3.9 pg/g and 4.3 pg/g TCDD for differing zones within the US. Other countries have set lower guideline levels, e.g., Finland (500 pg/g TEQ), Canada (350 pg/g TEQ) and Sweden (10 pg/g TEQ). For agricultural soil, the standard/guideline values are usually <= 10 pg/g, with the German standard being 5 pg/g; and the standard in England, Columbia, Canada, and Netherlands being 10 pg/g TEQ [3].

Three separate federal or international agencies have established a “safe” or tolerable daily dose for dioxin. In 1994, EPA defined an dose of 0.01 pg TEQ/kg bw/day, equivalent to 0.7 pg/day for a 70 kg adult, as posing a cancer risk of one additional cancer in one million people exposed [27]. This “risk dose equivalent” is designed to protect adults and does not include any added protection for children. In 1990, WHO established a tolerable daily intake (TDI), which ranged from 1 to 4 pg/kg bw/day for a daily ingestion of 70 to 280 pg in a 70 kg adult [27]. The Agency for Toxic Substances and Disease Registry assessed the non-cancer risks from dioxin exposure by setting minimal risk levels (MRLs) for acute, sub-chronic, and chronic exposures to dioxins. The chronic MRL was based on dioxin's developmental neurotoxicity in rhesus monkeys, and was set at 1 pg/kg bw/day [12].

## 4. Exposure Assessment

Local residents can be exposed to dioxin through oral, dermal and inhalation routes. However, it is believed that dioxin in foods is currently the major source of exposure [8]. Residents of Trung Dung and Tan Phong wards are exposed to dioxin in local foods, especially free-range chicken, duck, fresh fish and beef [8]. Although plant roots do not normally absorb dioxin [16,17], the consumption of vegetables and crops grown in and around the Bien Hoa Airbase may also lead to a dioxin exposure through food since dioxin in soil particles can attach to vegetables and crops and they may not be washed thoroughly. For infants, breast-feeding in the first few weeks after birth may also present a risk of dioxin exposure if the mother has been exposed to dioxin [28].

According to a study on levels of dioxin in foods at Bien Hoa City, a marked elevation of TCDD were reported in some of the food products, including ducks with 276 ppt and 331 ppt wet weight, chickens from 0.031–15 ppt wet weight, fish from 0.063–65 ppt wet weight, and a toad with 56 ppt wet weight, while the usual TCDD levels in food were less than 0.1 ppt [8]. However, it should be noted that in this study, 16 food samples taken at Bien Hoa Airbase, Bien Hung Lake and Bien Hung Market (in the vicinities of the Airbase) were analyzed, but these were not considered to be representative of foods consumed in the Trung Dung and Tan Phong wards nor in Bien Hoa City in general. Many types of foods being sold at these wards are not local foods, but imported from other areas where dioxin levels are believed to be much lower. Nevertheless, our survey of 400 households in the two wards showed that 40 households (10%) raised poultry, cattle or grew crops and vegetables in the last year, among them 27 households grew vegetables (16 households grew for their own consumption and 11 households for grew at large scale for sell) [30]. As such, if the dioxin levels in soil in the vicinity of the Bien Hoa Airbase were still high, the dioxin levels in these local foods are also believed to be high.

The WHO’s recommended tolerable daily intake (TDI) for a person weighting 70 kg ranged from 1 pg to 4 pg/1 kg body weight/day or (70 pg to 280 pg/day) [29]. Therefore, an adult Vietnamese person weighting an average of 50kg could be considered to have a TDI of 50 to 200 pg/day. If ducks with TCDD levels of 276 pg/g and 331 pg/g [8] were the sole source of dioxin exposure for people living near Bien Hoa Airbase, it could be calculated that the daily intake of duck resulting in a tolerable level of TCDD for an adult weighting 50 kg would be approximately 0.15–0.6 g duck/day if the duck had a TCDD level of 331pg/g (based on the calculations of [50 pg dioxin/person/day] / [331 pg dioxin/g duck] = 0.15 g duck/day to [200 pg dioxin/person/day] / [331 pg dioxin/g duck] = 0.6 g duck/day). Similar calculations can be made for other foods, as shown in the Table 2.

It should be noted that local people in the vicinity of Bien Hoa Airbase and in Trung Dung and Tan Phong wards have been exposed to dioxin in more than one type of food if they consume local products, and they may also be exposed to dioxin through dermal and inhalation routes. For example, our survey showed that 27 respondents (6.8%) reported having direct contact with soils and sediments regularly as part of their daily employment [30].

**Figure 1.**
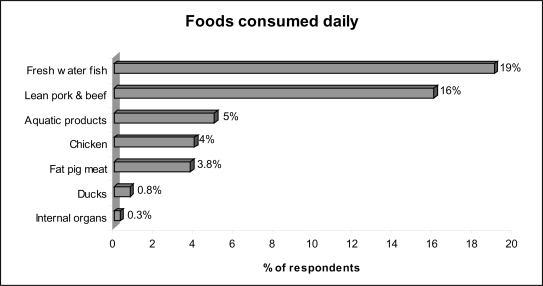
Foods consumed daily by local residents at Trung Dung and Tan Phong wards, Bien Hoa City [28].

**Figure 2.**
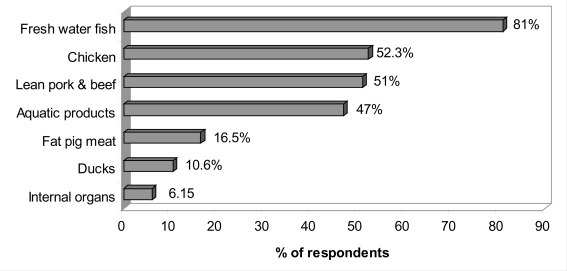
Foods consumed weekly by local residents at Trung Dung and Tan Phong wards, Bien Hoa City [28].

In reality, results of our food consumption frequency survey of 400 households randomly selected at Trung Dung and Tan Phong wards showed that high risk food such as fresh water fish, ducks, and chicken were usually presented in daily meals of local residents (see Figures 1 & 2) [30]. For example, 19% (CI 15.2%*–*22.9%) of respondents consumed fresh water fish daily. On a weekly basis, fresh water fish was consumed most frequently by 81% (CI 77.2%*–*84.8%) of respondents, followed by chicken 52.3% (47.4%*–*57.2%), lean pork meat and beef 51% (46.1%*–*55.9%), and other aquatic products (such as fresh water shrimp, snail, crab, *etc.*) 47% (CI 42.1%*–*52%). Ducks were consumed weekly by 10.6% (CI 7.6%*–*13.6%) households and animal viscera were consumed less frequently by 6.1% (CI 3.8%*–*8.5%) of residents. Therefore, based on the calculated average daily intake of dioxin from all sources (including all types of foods, dermal and inhalation exposures), residents of Trung Dung and Tan Phong wards, Bien Hoa City who consume local foods would far exceed the TDI recommended by WHO.

In two other studies, approximately 95% of blood samples taken from 43 accessible volunteers of various ages who lived near Bien Hung Lake (close to Bien Hoa Airbase) and who presumably consumed fish from the Lake were found to have elevated TCDD levels (above 5 ppt) [5,7]. These levels were greater than TCDD levels of less than 2 ppt reported in one pooled sample (n = 100) from North Vietnam (where no Agent Orange was sprayed or stored) [5]. Schecter *et al.* also reported a family whose members were heavy consumers of fish from Bien Hung Lake. All members had elevated TCDD levels: 271 ppt in the mother, 164 ppt in the father, and 87 ppt in a child born in 1980 (note that the spraying of Agent Orange ended early in 1971) [5]. There was another family who moved from northern Vietnam to Bien Hoa after Agent Orange spraying ended in 1971 and they also exhibited elevated TCDD levels, including TCDD levels of 57 ppt and 62 ppt in twin boys born in 1981, and their parents’ were 68 ppt and 74 ppt [7]. Clinical experience has shown that levels above 10 ppt are abnormal and can be harmful for health [3].

## 5. Risk Characterization

A review of biological properties and toxic effects of dioxins (TCDD in particular) indicates that this chemical is a carcinogen and a systemic toxicant capable of causing a significant range of health effects including chronic lymphocytic leukemia, soft-tissue sarcoma, non-Hodgkin’s lymphoma, Hodgkin’s disease, and chloracne [4]. There is also “limited or suggestive” evidence for an association between exposure to dioxin and laryngeal cancer, cancer of the lung, bronchus, or trachea, prostate cancer, multiple myeloma, AL amyloidosis, early-onset transient peripheral neuropathy, porphyria cutanea tarda, hypertension, Type 2 diabetes (mellitus), and spina bifida in offspring of exposed people [4].

A number of studies have reported elevated dioxin levels in samples of soil, sediment, various types of local foods and blood samples of local residents within the vicinity of Bien Hoa Airbase [3–6]. Even though there is no safe dose for dioxin exposure, the WHO has recommended a tolerable daily intake (TDI) for a person weighting 70 kg of between 1 pg and 4 pg/kg body weight/day [2,29]. Based on the calculations presented in this paper, an extremely small amount of local food products (such as fresh water fish, duck, free-range chicken) would need to be consumed by local residents to meet this TDI. Unfortunately, our food frequency survey of 400 randomly selected households at Trung Dung and Tan Phong wards showed that these potentially high risk foods were often consumed on a daily basis by local residents, as such, posing a high level of risk to health for these residents [30]. In addition, those households at Trung Dung and Tan Phong wards who consume self-cultivated foods would be at a very high risk as their daily intake of dioxin would far exceed the TDI recommended by the WHO.

There are some limitations to the risk assessment presented in this paper. Firstly, the exposure assessment is based on a range of studies, in which two studies had obtained a limited number of samples (16 food samples, 43 blood samples) that may not fully represent the situation in Bien Hoa City [7,8]. In addition, currently, there is still lack of data on dioxin levels in all major types of foods (both locally produced and imported) consumed in Trung Dung and Tan Phong wards as well as on the daily and weekly food consumption patterns of local residents. This information, together with information on dioxin levels in air, soil and water are needed to undertake complete exposure assessment calculations.

## 6. Stakeholder Engagement, Risk Communication & Community Consultation

Throughout the implementation process of this environmental health risk assessment activity, a number of stakeholders have been involved, including the Office of National Steering Committee on Overcoming Consequences of Toxic Chemicals used by US During the War in Vietnam, the Vietnam Public Health Association and its branch-Dong Nai Public Health Association, Vietnam National Institute of Nutrition, related departments at Bien Hoa City, Dong Nai Agent Orange Victim Association, the People’s Committee at provincial, district and ward levels, and the community. A one day public consultation meeting was also held at Bien Hoa City to share the results of our study and to obtain feedback on the assessment.

For risk communication purposes, dioxin exposure in Vietnam is a particularly sensitive issue and the adverse findings of any study may have substantial ramifications at a political, social, and economic level for the City. Therefore, extreme care was taken in communicating the findings of this risk assessment, with particular emphasis that not all foods consumed were contaminated, only those being grown/raised at dioxin contaminated areas in the vicinity of the Bien Hoa Airbase.

## 7. Risk Management

Because of its high potential to cause adverse health impacts in exposed populations, a number of activities have been undertaken to treat and contain dioxin contaminated soil at the Bien Hoa Airbase [4]. In addition, studies have now shown that dioxin is not only present in the soil of the Airbase but is also present at elevated levels in soil, water and foods of the surrounding areas [3–6,8,12]. Therefore, since early 2008, based on the result of this risk assessment, a multi-approach public health intervention program was developed and implemented, and this has been the first public health intervention program ever to be implemented in Vietnam to reduce the risks of dioxin exposure through foods for local residents surrounding the Bien Hoa Airbase. Details of this risk management intervention program and its results will be addressed in a separate paper.

## Figures and Tables

**Table 1.** Some Sensitive Endpoints of Dioxin Exposure [25,26].

**Species**	**Effect**	**Dose**	**Incremental Body Burden** (ng/kg)
Rhesus	Object learning	∼160 pg/kg/d	42*
Rhesus	Endometriosis	∼160 pg/kg/d	42**
Rat	Genital malformation	200 ng/kg #	73*
Rat	Immune suppression	100 ng/kg #	50*
Rat	Decreased sperm count	64 ng/kg #	28*
Mouse	Immune suppression (viral susceptibility)	10 ng/kg #	10
Current Average Body Burden Levels in Humans (“Background”)			∼10

Notes: Rodent background body burdens are about 4 ng/kg; # Single dose on specific day of pregnancy;

* Estimated maternal body burden above background;

** Estimated body burdens above background.

**Table 2.** Calculated amount of daily food intake that is tolerable for a local person weights 50 kg if each type of food is the only source of dioxin exposure.

**Types of foods in Bien Hoa**	**Levels of TCDD contamination** (ppt or pg/g)*	Approximate daily food intake (g) that is tolerable for person weighing 50 kg**
Duck/wild goose	276*–*331	0.15*–*0.6 g
Duck/wild goose fat	536–550	0.09*–*0.36 g
Snakehead fish	66	0.76*–*3.0 g
Snakehead fish fat	15,349	0.003*–*0.013 g
Chicken	0.35*–*48	1–4 g
Toad	80	0.63–2.5 g
Toad fat	11,765	0.004–0.017 g
Pig	0.6*–*1.1	45–180 g
Beef	0.11*–*0.21	238–950 g

Notes:

* = samples were taken at Bien Hoa Airbase, Bien Hung Lake and Bien Hung Market [8];

** = the food intakes were calculated based on the assumption that each type of food was the only source of dioxin exposure. In practice, local people have been exposed to dioxin in a variety of types of foods and also through other exposure routes (such as dermal and inhalation), therefore the amount of each type of foods to be consumed should be lower than values calculated in this table in order for the local residents to meet TDI level.
